# Genome-wide transcriptomic analysis of a desert willow, *Salix psammophila*, reveals the function of hub genes *SpMDP1* and *SpWRKY33* in drought tolerance

**DOI:** 10.1186/s12870-019-1900-1

**Published:** 2019-08-15

**Authors:** Huixia Jia, Jin Zhang, Jianbo Li, Pei Sun, Yahong Zhang, Xuebing Xin, Mengzhu Lu, Jianjun Hu

**Affiliations:** 1 State Key Laboratory of Tree Genetics and Breeding, Key Laboratory of Tree Breeding and Cultivation of National Forestry and Grassland Administration, Research Institute of Forestry, Chinese Academy of Forestry, Beijing, 100091 China; 2 Biosciences Division, Oak Ridge National Laboratory, Oak Ridge, TN 37831 USA; 3 Experimental Center of Forestry in North China, Chinese Academy of Forestry, Beijing, 102300 China

**Keywords:** *Salix psammophila*, Drought, Co-expression network, Hub gene, Transgene, *SpMDP1*, *SpWRKY33*

## Abstract

**Background:**

Drought is a major environmental constraint to plant growth, development and productivity. Compared with most willows that are generally susceptible to drought, the desert willow *Salix psammophila* has extraordinary adaptation to drought stress. However, its molecular basis of drought tolerance is still largely unknown.

**Results:**

During polyethylene glycol 6000 (PEG 6000)-simulated drought stress, we found that the osmotic adjustment substances were accumulated and the antioxidant enzyme activities were enhanced in *S. psammophila* roots. A total of 8172 differentially expressed genes were identified in roots of *S. psammophila* through RNA-Sequencing. Based on K-means clustering, their expression patterns were classified into nine clusters, which were enriched in several stress-related processes including transcriptional regulation, response to various stresses, cell death, etc. Moreover, 672 transcription factors from 45 gene families were differentially expressed under drought stress. Furthermore, a weighted gene co-expression network was constructed, and eight genes were identified as hub genes. We demonstrated the function of two hub genes, *magnesium-dependent phosphatase 1* (*SpMDP1*) and *SpWRKY33*, through overexpression in *Arabidopsis thaliana*. Overexpression of the two hub genes enhanced the drought tolerance in transgenic plants, suggesting that the identification of candidate drought tolerance genes in this study was highly efficient and credible.

**Conclusions:**

Our study analyzed the physiological and molecular responses to drought stress in *S. psammophila*, and these results contribute to dissect the mechanism of drought tolerance of *S. psammophila* and facilitate identification of critical genes involved in drought tolerance for willow breeding.

**Electronic supplementary material:**

The online version of this article (10.1186/s12870-019-1900-1) contains supplementary material, which is available to authorized users.

## Background

Forest ecosystems account for ~ 30% of the land surface (covering ~ 42 million km^2^), providing many benefits to natural systems and humankind from ecological, economic, social, and aesthetic services [1, 2]. Drought is a major environmental constraint to plant growth, development and productivity. Notably, owing to the long-life spans of forest tree and difficulty for implementing irrigation on a large scale, drought greatly reduces tree productivity and survival across many forest ecosystems [3]. The research of current climate models has predicted increased drought episodes due to the long-term effects of global warming [4]. Therefore, expediting dissection of drought tolerance mechanism and improving drought adaptation of forest trees are urgent needed for arid land utilization, environmental sustainability and improvement of economic benefits.

To accommodate drought stress, plants have evolved sophisticated mechanisms to perceive external signal and coordinate metabolic pathways and morphological traits by modulating the expression of genes [5]. Currently, many drought-inducible genes have been identified through transcriptome analysis in *Arabidopsis* [6–8], rice [9–11], maize [12], and other species. These genes can be classified into two classes: functional genes and regulatory genes [5]. Overexpression of some drought-responsive genes (e.g., *ethylene response factor 48*, *calcium-dependent protein kinase 4*, *NAC5*, *MYB5*, etc.) has demonstrably enhanced drought tolerance in transgenic plants [13–16]. Nevertheless, large variations occur in transcriptional responses to drought among different plant species [17, 18]. Most researches of drought responses mechanisms have concentrated in crops or model species. Research into forest trees is still at an initial stage except several species, such as *Populus*, *Pinus*, *Eucalyptus* and *Quercus* [19, 20]. Thus, studies on the species-specific molecular mechanisms of drought response are essential for molecular breeding for drought tolerance in forest trees.

Willows (*Salix*; Salicaceae) are a very diverse group of dioecious catkin-bearing trees and shrubs, which were the research focus as a potential source of sustainable and renewable biomass for bioenergy, biofuels and bioproducts [21, 22]. However, willows are generally susceptible to drought stress, so they commonly grow in alluvial or riparian habitats [23]. In recent years, willow regulatory responses to drought at morphological, physiological, genomic and transcriptional levels have been examined in some studies, and some loci or candidate genes associated with drought responses were identified. For example, quantitative trait loci (QTLs) associated with drought tolerance for *Salix dasyclados* × *S. viminalis* hybrid in two water regimes (normal and drought-treated) have been identified, and each QTL explained 8 to 29% of the phenotypic variation [24]. Fifteen willow genotypes are assessed the impact on the growth and leaf traits under permanent and temporary water stresses, and great variation in drought response among genotypes has been found [25]. Two willow genotypes that originated from a cross between *S. viminalis* and *S. viminalis* × *S. schwerinii* and displayed different responses to drought have been performed phenotypic and transcriptional investigation under drought stress, and a set of candidate genes associated with drought response have been identified [18]. Despite these advances, the comprehensive molecular mechanisms of drought resistance in willows remain poorly elucidated, and functional validation for detected candidate genes has not been carried out.

In willows, *S. psammophila* is an important shrub biomass willow that distributed in arid and semi-arid desert regions. It can well adapt to multiple abiotic stresses including drought, extreme temperature, sandstorm, and so on [26]. So, it is used to prevent wind erosion and control desertification, playing vital roles in local vegetation rehabilitation [27]. Furthermore, it is identified as a promising biomass feedstock [28, 29]. Drought tolerance plants that can maintain turgor and continue metabolism in cells even at low water potential are identified as potential sources for studying the drought tolerance mechanisms and exploring key regulatory genes for breeding [30]. However, the drought response mechanism of *S. psammophila* is still poorly understood.

The aim of this study is to investigate the drought response mechanism and identify key regulatory genes in *S. psammophila*. In order to achieve this, physiological responses to drought stress were analyzed. RNA-Sequencing technology was used to identify differentially expressed genes (DEGs) of root tissue of *S. psammophila* at different time points (0, 6, 24 and 96 h) after drought treatment. Enrichment analysis was performed to identify pivotal functional categories involved in drought response. Based on the DEGs, gene co-expression network was constructed to identify hub genes associated with drought tolerance, and two hub genes were selected for functional verification through overexpression in *A. thaliana*. Our results contribute to a better understanding of drought response in *S. psammophila*; and suggest that co-expression network has important potential to facilitate the identification of critical genes involved in drought tolerance.

## Results

### Physiological responses to drought stress in *S. psammophila*

**Fig. 1:**
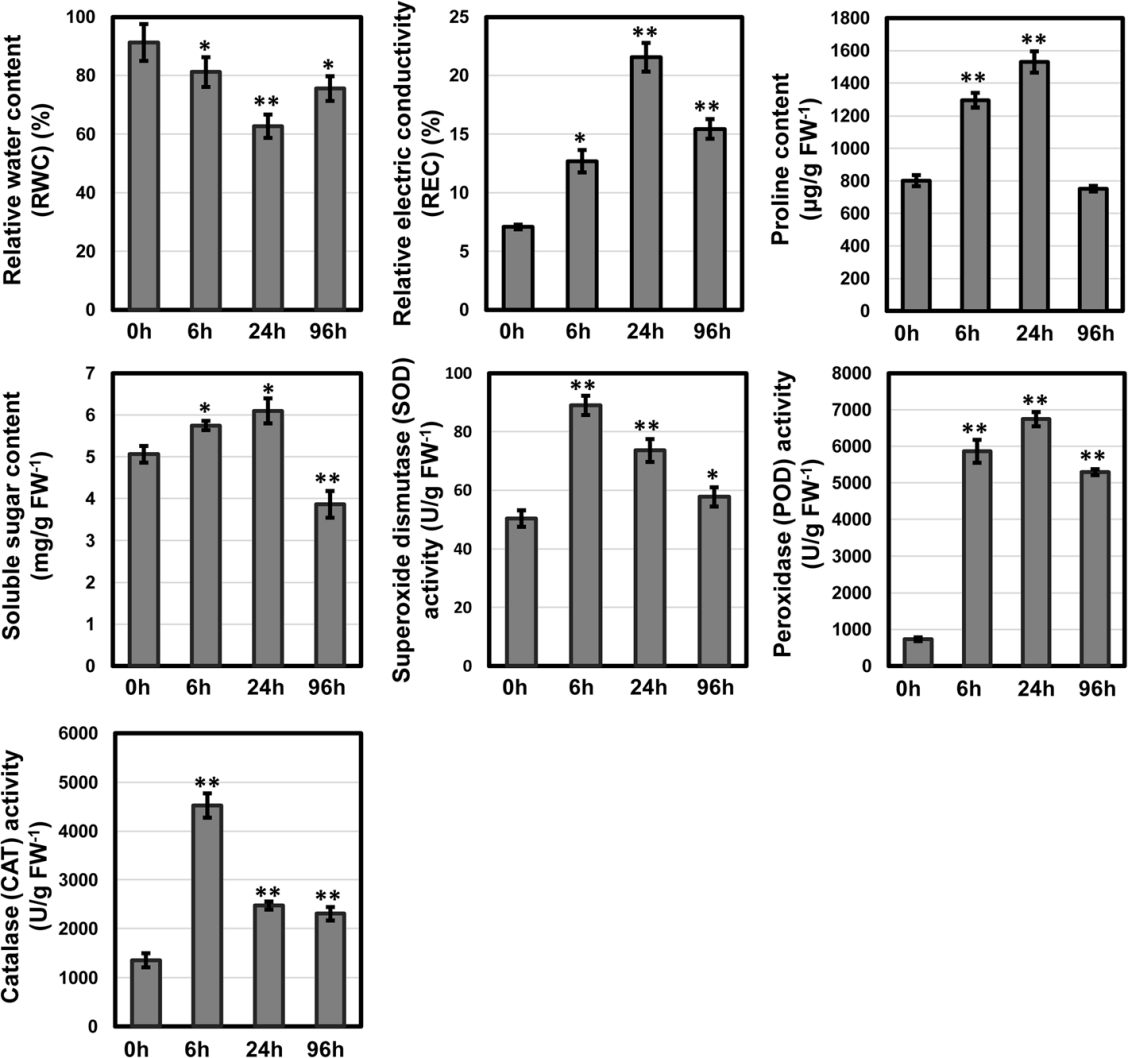
Physiological responses to drought stress in the roots of *S. psammophila*. The experiments were performed in three biological replicates and three technical replicates. The data were analyzed with Student’s *t* test. * and ** represent significance of *P* < 0.05 and *P* < 0.01 of three time points (6, 24 and 96 h) compared with the control (0 h), respectively

To explore the drought tolerance mechanism of *S. psammophila*, fine roots at four time points (0, 6, 24 and 96 h) under 22% (w/v) polyethylene glycol 6000 (PEG 6000)-simulated drought stress were used for both physiological and molecular experiments. To avoid rhythm affects and increase data comparability, the different treatments were started at corresponding time points prior to the harvest time and all the samples were collected at the same time [31]. We measured relative water content (RWC), relative electric conductivity (REC), contents of osmotic adjustment substances (proline and soluble sugars) and activities of antioxidant enzymes (superoxide dismutase, peroxidase and catalase) of roots to evaluate the physiological responses to drought stress. The RWC was detected as a measure of the ability of plants to absorb water, and it exhibited striking decrease (*P* < 0.01) continually from 0 h to 24 h, following slight increase at 96 h (Fig. 1). Interestingly, the REC, osmotic adjustment substances and antioxidant enzyme activities showed opposite trend of initially increase and then decrease during drought stress (Fig. 1).

### RNA-Sequencing profiling and global transcriptome changes during drought stress in *S. psammophila*

**Fig. 2:**
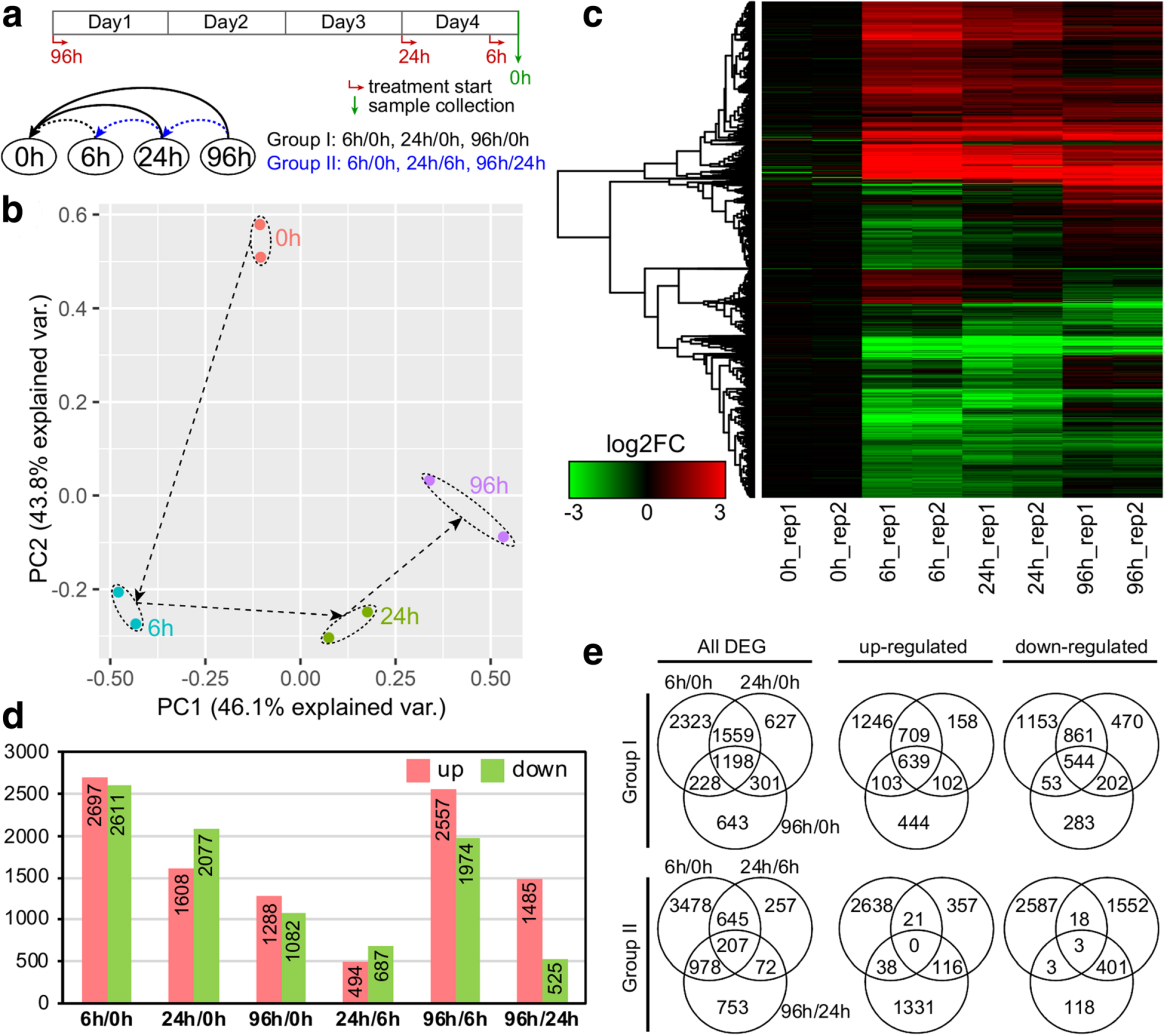
Transcriptome analysis of differentially expressed genes (DEGs) during the drought stress in the roots of *S. psammophila*. **a** Harvest regime designed for a treatment-versus-control experiment with four time points (0, 6, 24 and 96 h) after 22% (w/v) polyethylene glycol 6000 (PEG 6000)-simulated drought stress. All treatments were started at different calculated times prior to the harvest day, and all root samples were designed to be harvested at the same time. **b** Principal component analysis (PCA) showing the uniformity between biological replicates and the relationships of samples at four stages. The PC1 and PC2 explaining 46.1 and 43.8% of the total variance, respectively. **c** Hierarchical cluster analysis of total DEGs. Columns and rows in the heat maps represent samples and genes, respectively. **d** The number of up- and down-regulated genes during drought stress with respect to the control or the preceding time point. **e** Venn diagram of DEGs showing common or uniquely regulated genes at different time points during drought stress

To further elucidate the molecular basis for drought responses in *S. psammophila*, a global transcriptomic analysis was performed using RNA-Sequencing. cDNA libraries with two biological replicates at each time point were independently constructed from *S. psammophila* roots harvested at the four time points (0, 6, 24 and 96 h) after drought treatment (Fig. 2a), and subsequently sequenced using the Illumina HiSeq2500 platform. After quality control of raw sequencing reads, a total of 297,112,106 clean reads (37.43 Gb) with paired-ends of 125 bp were retained for further analysis. The GC content was 44.23% and the Q30 was 92.34% (Additional file 1).

Approximately 83.50% of total reads were aligned to *S. purpurea* reference genome version 1.0, a well assembled *Salix* genome until now (Additional file 1). Principal component analysis (PCA) was carried out on gene expression dataset to confirm the uniformity between biological replicates and investigate the relationships of samples at the four stages (Fig. 2b). Tight cluster between two biological replicates indicated the reliability of our RNA-Sequencing dataset, while greatest distinctness existed between 6 h samples and control (0 h) samples, with PC1 and PC2 explaining 89.9% of the total variance. Hierarchical clustering analysis supported these results (Fig. 2c).

DEGs were identified in different comparisons, which were classified into two groups: Group I for different treatment timepoints compare to control (6 h/0 h, 24 h/0 h and 96 h/0 h) and Group II in time-course manner (6 h/0 h, 24 h/6 h and 96 h/24 h) (Fig. 2a). The largest DEG set was identified in comparison “6 h/0 h” (a total of 5308 DEGs including 2697 up- and 2611 down-regulated genes), suggesting that *S. psammophila* roots rapidly reprogrammed cellular response in transcriptomic level at early stage (6 h) under drought stress. In contrast, the smallest DEG set was identified in comparison “24 h/6 h” (1181 DEGs including 494 up- and 687 down-regulated genes), followed by comparison “96 h/24 h” (2010 DEGs including 1485 up- and 525 down-regulated genes) (Fig. 2d). Comparative analysis revealed that the overlapped DEGs in three comparisons of Group I (1,198 common genes, 14.7% of total DEGs) were greater than that in Group II (207 common genes, 3.2% of total DEGs) (Fig. 2a and e). The greatest subset of stage-specific DEGs was in comparison “6 h/0 h” in both two groups (2323 DEGs in Group I and 3478 DEGs in Group II). In addition, less or no overlap of up-regulated and down-regulated genes in Group II (Fig. 2e).

### Functional annotation and pathway enrichment analysis of DEGs

**Fig. 3:**
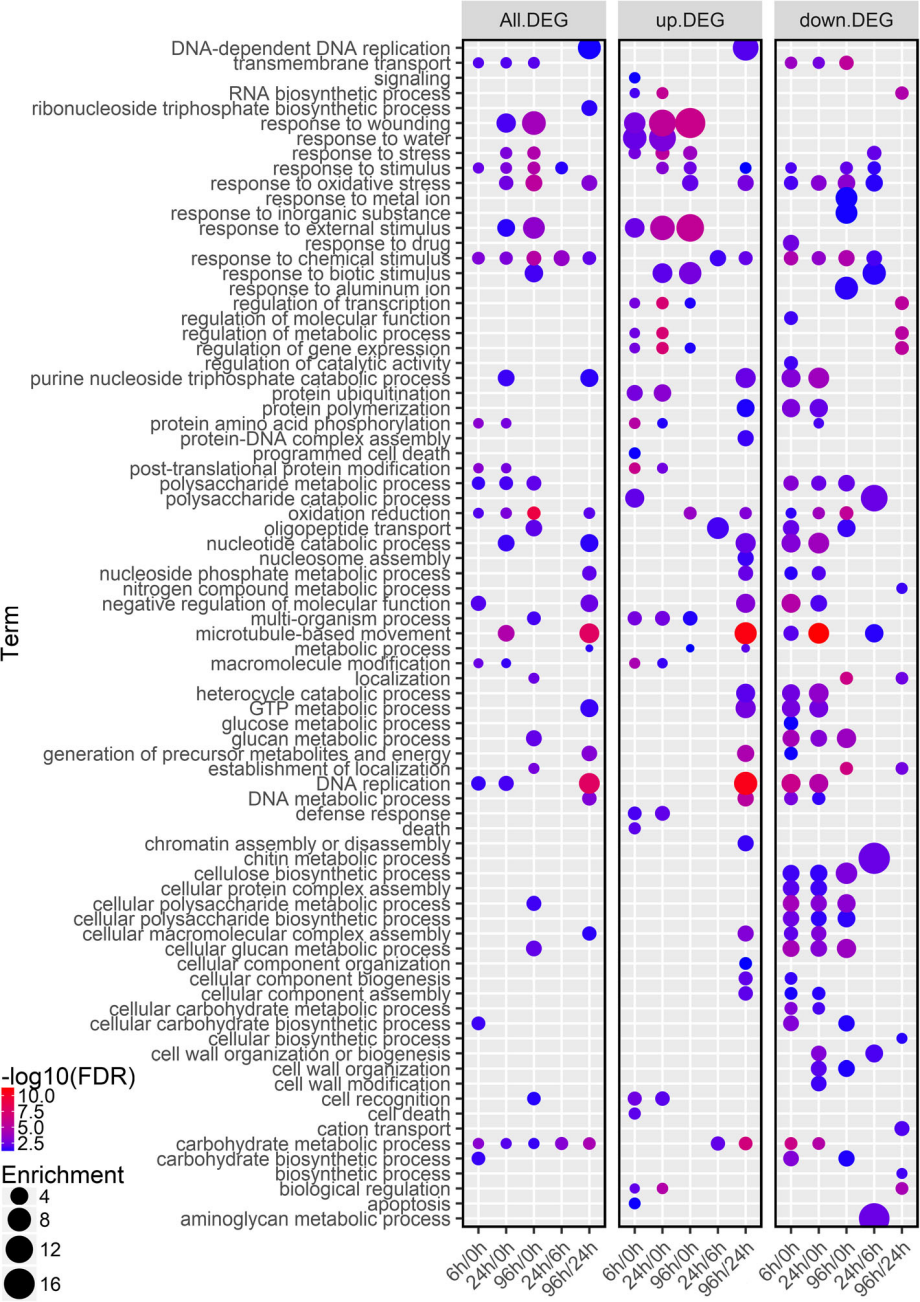
Biological processes in gene ontology (GO) enrichment analysis of DEGs during drought stress in *S. psammophila*. GO enrichment analysis was performed using Blast2GO program. Only significantly enriched terms with corrected *P* < 0.05 were indicated. The color and size of each point represented the -log_10_ (FDR) values and enrichment scores. A higher -log_10_ (FDR) value and enrichment score indicated a greater degree of enrichment

To identify the major functional categories represented by the DEGs, gene ontology (GO) enrichment analysis was performed. These DEGs comprised three major enrichment categories: 79 biological processes (BP) (Fig. 3), 112 molecular functions (MF) (Additional file 2: Figure S1), and 28 cellular components (CC) (Additional file 2: Figure S2 and Additional file 3). Up-regulated genes were enriched in 49 BP GO terms, including several stress-responsive processes (e.g., response to stress wounding, water, stress, external or biotic stimulus) that were prominent in Group I comparisons (6 h/0 h, 24 h/0 h and 96 h/0 h), but did not enrich in 24 h/6 h and 96 h/24 h (Fig. 3). Down-regulated genes were enriched in 58 BP GO terms: the GO terms of ‘multiple carbohydrates biosynthesis and metabolic process’ and ‘cell wall organization and modification’ were mainly enriched in Group I comparisons; the GO terms of ‘chitin metabolic process’ and ‘aminoglycan metabolic process’ were prominent only in 24 h/6 h (Fig. 3). In addition, GO terms of ‘response to stimulus’, ‘response to oxidative stress’, ‘response to chemical stimulus’, ‘microtubule-based movement’ and ‘DNA replication’ were prominent in all comparisons (Fig. 3).

In MF category, the GO terms such as ‘ubiquitin-protein ligase activity’, ‘serine-type endopeptidase inhibitor activity’, ‘peptidase inhibitor activity’, ‘endopeptidase inhibitor activity’ and ‘carbohydrate binding’ were mainly enriched in Group I comparisons (6 h/0 h, 24 h/0 h and 96 h/0 h) in up-DEGs; the GO terms of ‘polysaccharide binding’, ‘pattern binding’ and ‘chitin binding’ were specifically enriched in 96 h/0 h in up-DEGs. The GO terms such as ‘glucosyltransferase activity’, ‘copper ion binding’ and ‘cellulose synthase activity’ were enriched in Group I comparisons in down-DEGs (Additional file 2: Figure S1). In CC category, up-regulated genes were significantly enriched in GO terms of ‘photosystem’, ‘thylakoid’, ‘nucleosome’, ‘microtubule’, ‘chromosome’ and ‘cell wall’ mainly in 96 h/24 h; down-regulated genes in Group I comparisons were significantly enriched in GO terms of ‘periplasmic space’, ‘microtubule’, ‘membrane’, ‘external encapsulating structure’, ‘cell wall’, ‘cell envelope’, etc. (Additional file 2: Figure S2).

To gain further insight into the molecular response to drought of *S. psammophila* in pathway level, we performed an enrichment analysis based on the KEGG pathways. A total of 15 pathways were significantly enriched in this process (Additional file 2: Figure S3 and Additional file 4). In particular, pathways of ‘phenypropanoid biosynthesis’, ‘metabolism of xenobiotics by cytochrome P450’, ‘phenlylalanine metabolism’ and ‘glutathione metabolism’ were significantly enriched in most comparisons, and the degree of enrichment increased with time extension under drought treatment. The pathway of ‘nitrogen metabolism’ was significantly enriched in early and medium stages (6 h/0 h and 24 h/0 h); the pathway of ‘starch and sucrose metabolism’ was significantly enriched in early and late stages (6 h/0 h and 96 h/0 h); the pathway of ‘pentose and glucuronate interconversions’ was significantly enriched in medium and late stages (24 h/0 h and 96 h/0 h). These three pathways also prominent in comparisons 24 h/6 h and 96 h/24 h. Moreover, the pathways of ‘flavonoid biosynthesis’, ‘cell cycle’, ‘gap junction’, ‘tyrosine metabolism’, ‘cyanoamino acid metabolism’ and ‘alpha-linolenic acid metabolism’ were specifically enriched in different stages in Group I comparisons (Additional file 2: Figure S3).

### K-means clustering of DEGs

**Fig. 4:**
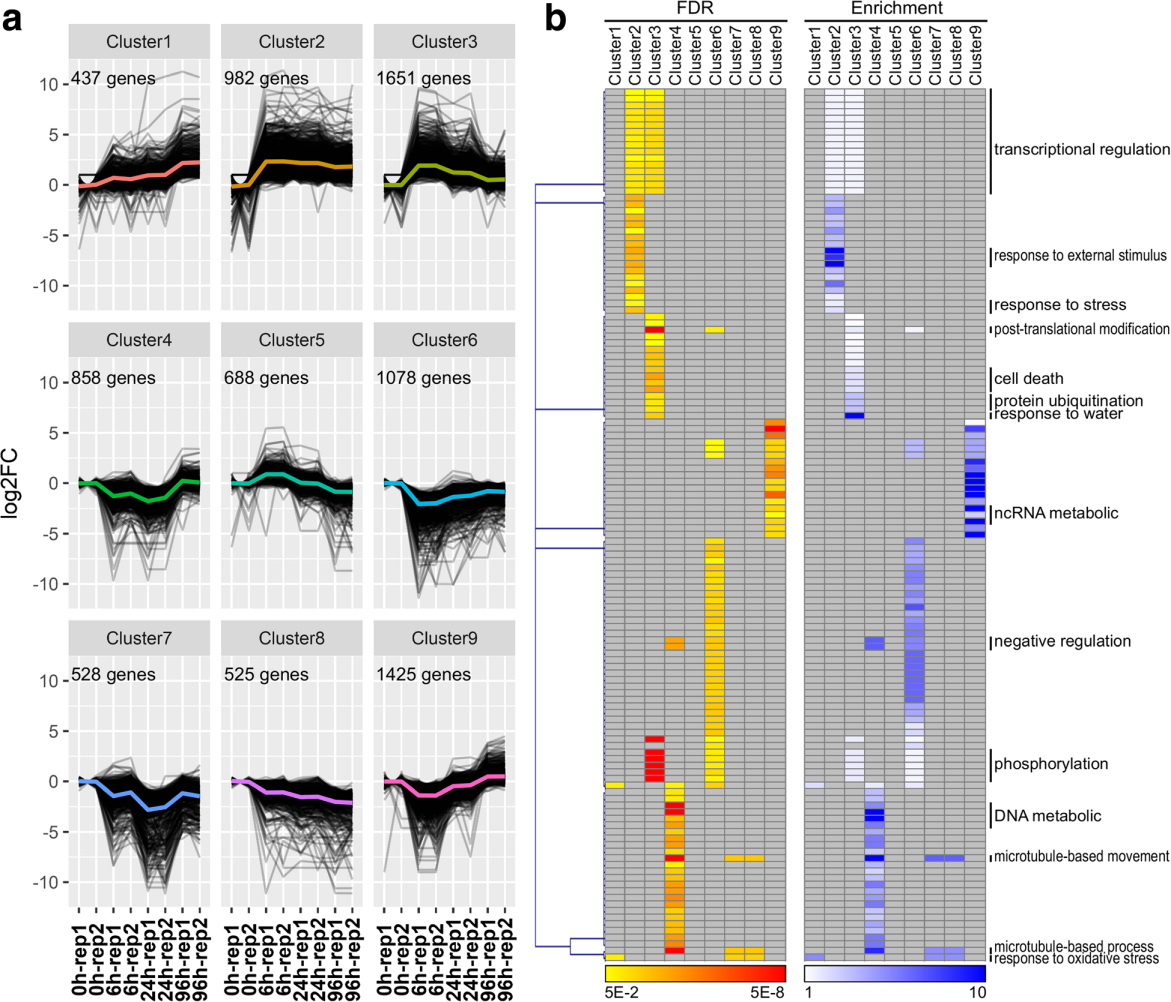
Cluster analysis of DEGs based on the K-means method and cluster-specific GO enrichment analysis. **a** K-means clustering analysis identified nine clusters of DEGs according to their expression patterns. Mean expression values of each cluster were highlighted. **b** Gene ontology (GO) terms enrichment analysis of nine DEG clusters

K-means clustering algorithm was applied to divide the 8172 DEGs into nine clusters with characteristic expression patterns and dynamics (Fig. 4a). Each cluster was distinctively composed of genes with particular biological functions, as highlighted by the cluster-specific GO enrichment (Fig. 4b and Additional file 5). In cluster 1, a total of 437 genes showed gradual up-regulation during the treatment and were enriched in ‘response to oxidative stress’ and ‘carbohydrate metabolic process’ terms. The expression of 982 genes in cluster 2 were dramatically induced at 6 h and maintained at an elevated level during drought stress, whereas 1651 genes in cluster 3 were abundantly expressed at early stage and then gradually reduced at 24 h and 96 h. Genes in both clusters 2 and 3 were enriched in transcriptional regulation-related GO terms. Notably, genes in cluster 2 were specifically enriched in ‘response to stress/external stimulus’; cluster 3 genes were enriched in the GO terms of ‘response to water’, ‘cell death’, ‘post-translational modification’, ‘protein ubiquitination’ and ‘phosphorylation’. The gene expression profiles were similar in clusters 4 (858 genes) and 6 (1078 genes), albeit with slightly different degrees of increase at 96 h. GO terms of ‘negative regulation’ were enriched in these two clusters, meaning negative regulation-related genes were inhibited during drought treatment. Compared with cluster 6, genes that encode proteins for the ‘DNA metabolic’ and ‘microtubule-based process and movement’ were highly enriched in cluster 4.

Transcription factors (TFs) act as a pivotal part of transcriptional regulation in plant development and stress response. We identified 672 TFs from 45 gene families were differentially expressed in the nine clusters (Additional file 2: Figure S4). The largest number of TFs were distributed in cluster 3 (164 TFs) and cluster 2 (109 TFs). TF families WRKY, NAC, MYB and ERF were abundant in these two clusters representing genes that were quickly induced under the early stage during drought stress (Fig. 4). Except for them, the members of LBD, HD-ZIP, bZIP, GRAS, G2-like, C2H2 and bHLH families were widely and unevenly distributed among the nine clusters (Additional file 2: Figure S4).

### Construction of co-expression network and identification of hub genes

**Fig. 5:**
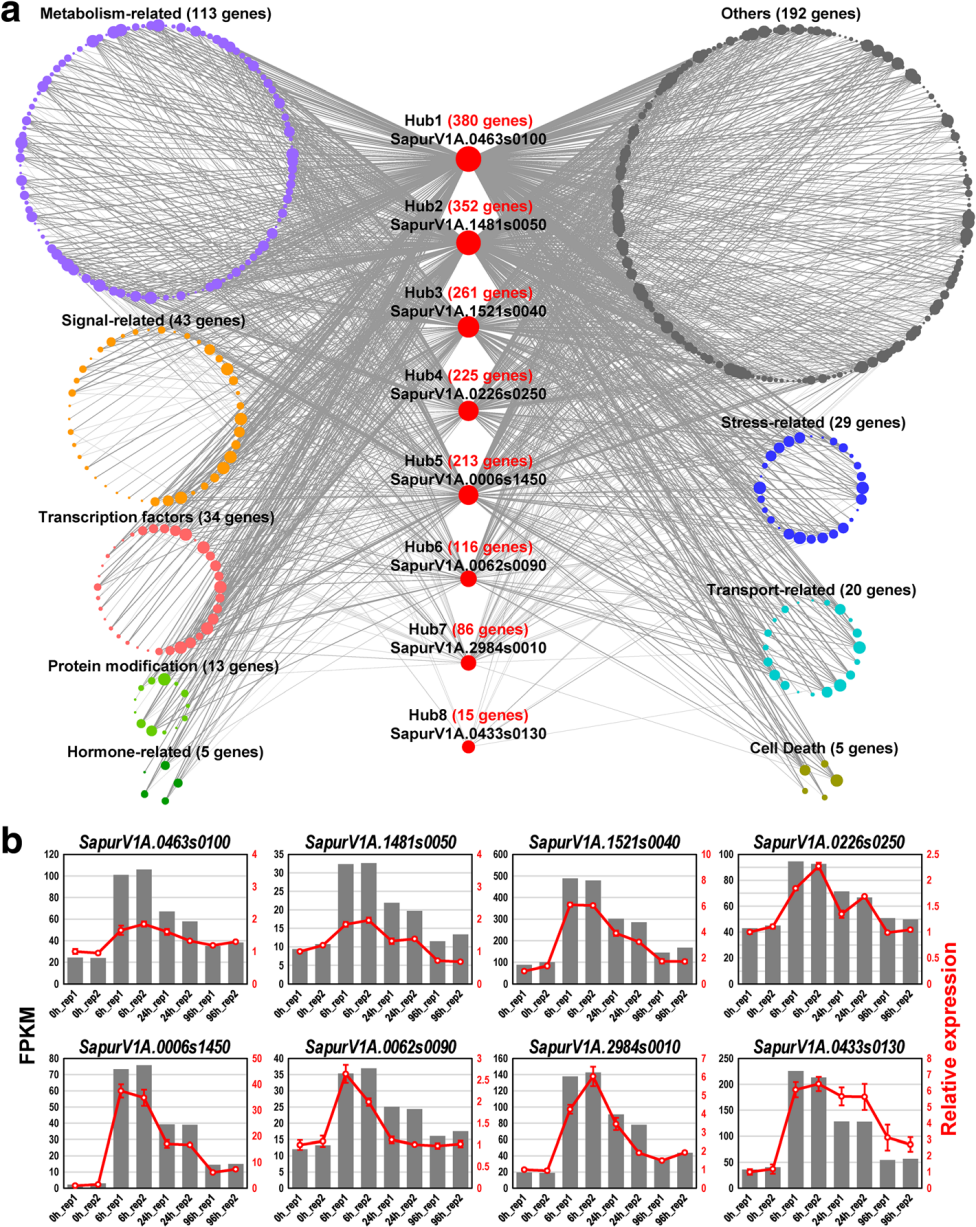
Identification of hub genes in co-expression network and their expression levels under drought stress. **a** Eight hub genes and their connectivity with co-expressed genes in the network. The co-expression network was analyzed using WGCNA software and the graphic network was created by Cytoscape software. This co-expression network was constructed from 464 genes from six co-expression modules (Additional file 2: Figure S5). **b** Expression confirmation of eight Hub genes using qRT-PCR. Each sample was conducted three biological replicates and four technical replicates. FPKM values of eight Hub genes from RNA-Sequencing at four time points were shown as grey columns, and relative expression of target genes by qRT-PCR were shown as red lines with standard error. Transcript level at control (0 h) was set to 1. Details of the 464 co-expressed genes was listed in Additional file 6

To investigate the interrelationships among drought-responsive genes and identify the key regulators, we constructed a co-expression network through weighted gene co-expression network analysis (WGCNA). According to the DEGs, their expression patterns were divided into six co-expression modules (Additional file 2: Figure S5a). Hierarchical clustering analysis clustered the six modules into three branches: module 1 and module 6, module 3 and module 4, module 2 and module 5 (Additional file 2: Figure S5b). Based on the co-expression relationships, eight genes with the highest connectivity values in this network were identified as hub genes (Fig. 5a), including *magnesium-dependent phosphatase 1* (*MDP1*, *SapurV1A.0463 s0100*), *RING domain ligase2* (*RGLG2*, *SapurV1A.1481 s0050*), *modifier of rudimentary* (*Mod*(*r*)) *protein* (*SapurV1A.0226 s0250*), *calmodulin-like 8* (*CML8*, *SapurV1A.0006 s1450*), *receptor-like cytoplasmic kinase* (*RLCK*, *SapurV1A.0062 s0090*), *GRAM domain-containing protein* (*SapurV1A.2984 s0010*), transcription factor *WRKY DNA-binding protein 33* (*WRKY33*, *SapurV1A.0433 s0130*) and an unknown gene (*SapurV1A.1521 s0040*). These genes displayed connectivity with 456 co-expressed genes, which were classified into nine categories according to their functional annotation: 113 metabolism-related genes, 43 signal-related genes, 34 transcription factors, 29 stress-related genes, 20 transport-related genes, 13 protein modification genes, 5 hormone-related genes, 5 cell death-related genes and 192 unclassified genes (Fig. 5a and Additional file 6). The functional classification of co-expressed genes including eight hub genes was performed based on the functional annotation. The expression profiles of the eight hub genes showed rapidly increase at 6 h, and gradually decline but were higher than the control at 24 h and 96 h (Fig. 5b). These eight hub genes were regarded as promising candidate genes for drought tolerance.

### Overexpression of hub genes *SpMDP1* or *SpWRKY33* confers drought tolerance in *Arabidopsis*

**Fig. 6:**
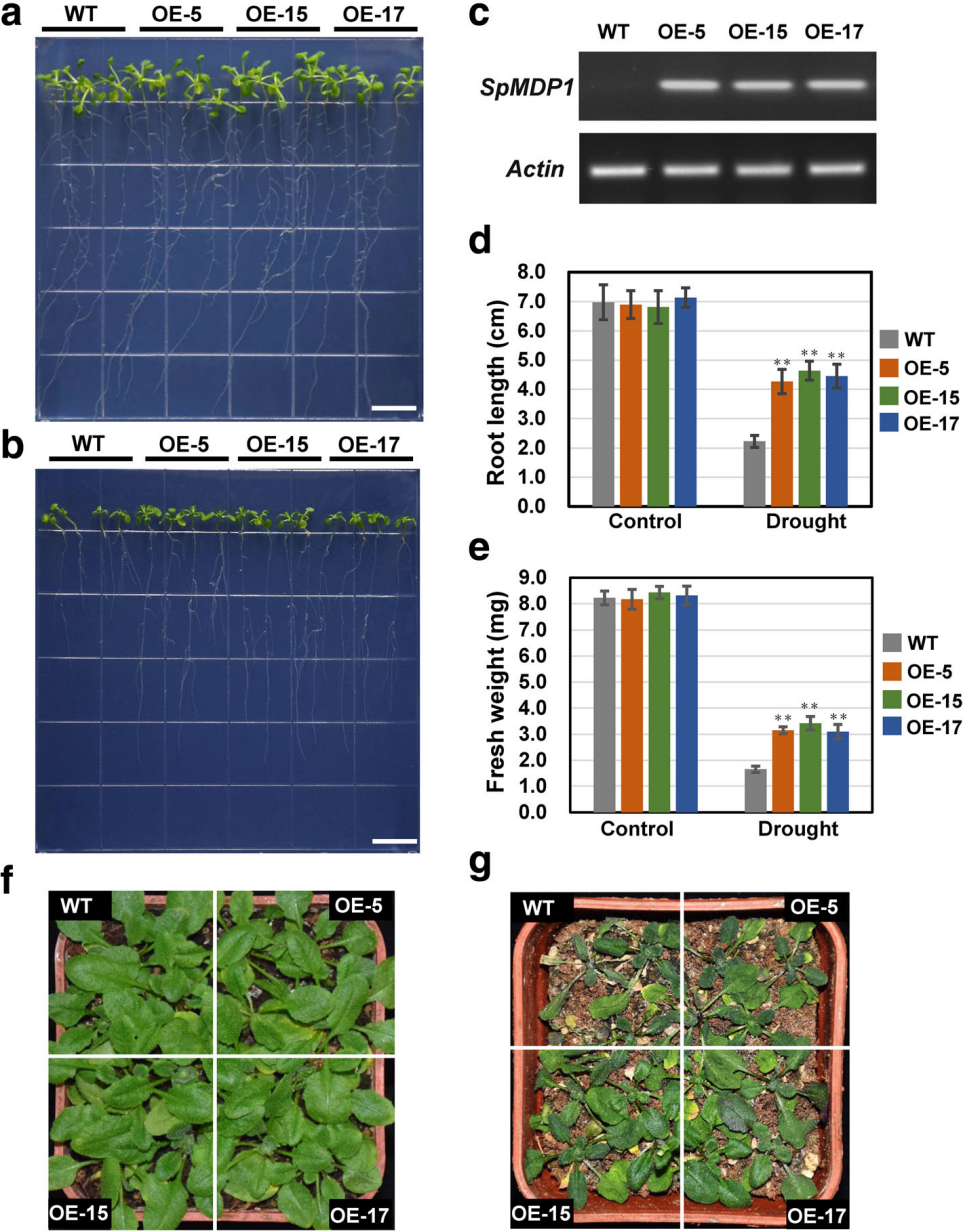
Overexpression of *SpMDP1* enhanced drought tolerance in transgenic *Arabidopsis* plants. **a** and **b** Phenotypic characteristics of wild type (WT) and three independent *SpMDP1*-overexpression lines (OE-5, OE-15 and OE-17) in control and mannitol-simulated drought treatment for 10 days, respectively. WT and three independent transgenic lines were plated in per plate, with four seedlings of each genotype. The drought treatments in petri plate were performed five biological replicates. Scale bar: 1 cm. **c** Semi-quantitative RT-PCR analysis of *SpMDP1* expression in WT and transgenic plants. **d** and **e** Statistics of root length and fresh weight of WT and *SpMDP1*-overexpression plants, respectively. Values were means ± SD (*n* = 20). ** on the histograms indicates statistical difference between WT and transgenic plants at *P* < 0.01. **f** and **g** Phenotypic characteristics of WT and *SpMDP1*-overexpression plants under normal water condition and withholding watering for 14 days, respectively. WT and three independent transgenic lines were grown in soil block, with four seedlings of each genotype. The drought treatments in soil were performed five biological replicates

To verify the reliability of hub genes identified through WGCNA, we selected two hub genes (*SpMDP1* and *SpWRKY33*) that displayed high connectivity and low connectivity respectively in eight hub genes to conduct functional verification through overexpression in *A. thaliana*. T3 generation of three independent transgenic *Arabidopsis* lines (OE-5, OE-15 and OE-17) with high abundance of *SpMDP1* and wild type (WT) were chosen to assess their drought tolerance (Fig. 6a-c). In petri plate, the root length and fresh weight of transgenic lines were significantly higher than that of WT under drought stress simulated by mannitol (200 mmol/L), but no significant difference was observed between WT and transgenic lines under control condition (Fig. 6d-e). In soil drought treatment, the transgenic lines also showed stronger drought tolerance than WT (Fig. 6f-g). These results of two parallel experiments both indicated that *SpMDP1* improved the tolerance to drought stress in transgenic *Arabidopsis*.

In the co-expression network, *SpMDP1* had the highest connectivity with 380 genes, most of which have previously been reported to participate in drought response or improve drought tolerance, for instance, several transcription factors (*ERF*, *bHLH*, *WRKY*, *C2H2*, *bZIP*, *MYB*, *NAC*, etc.), *late embryogenesis abundant* (*LEA*) *hydroxyproline-rich glycoprotein* (*LEA-HRGP*), *CBL-interacting protein kinase*, *mitogen-activated protein kinase* (Additional file 7). The *Arabidopsis* homologous genes of *SpMDP1*-co-expressed genes were searched using sequences alignment (Additional file 7). To validate the relationship of *SpMDP1* and its co-expressed genes, the expression patterns of 30 randomly selected *Arabidopsis* homologous genes in the co-expression sub-network of *SpMDP1* were compared between two-week-old *SpMDP1*-overexpressing plants and WT plants. Among them, the expression levels of 29 genes were unambiguously detected: 13 genes were up-regulated, four genes were down-regulated, and the expression levels of other 12 genes did not change (Additional file 2: Figure S6). Of the up-regulated genes, most genes were up-regulated by > 2-fold in transgenic plants, such as *WRKY75* (2.3- to 3.2-fold), *LEA-HRGP* (2.1- to 3.1-fold), *HSP17.6* (3.2- to 4.5-fold), *GSTU25* (3.6- to 4.8-fold), and *NAC90* (18- to 24-fold) (Additional file 2: Figure S6).

**Fig. 7:**
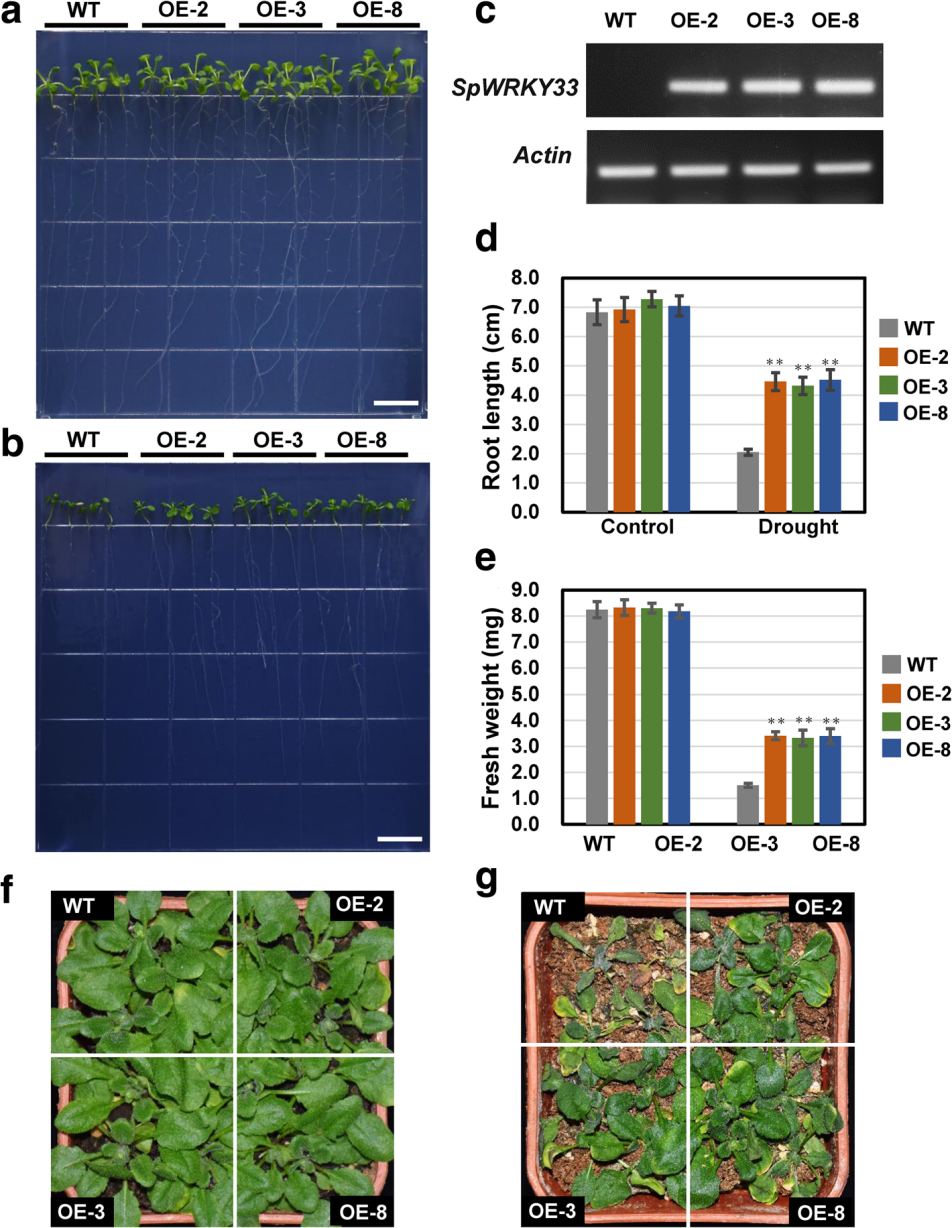
Overexpression of transcription factor *SpWRKY33* enhanced drought tolerance in transgenic *Arabidopsis* plants. **a** and **b** Phenotypic characteristics of wild type (WT) and three independent overexpression lines (OE-2, OE-3 and OE-8) in control and mannitol-simulated drought treatment for 10 days, respectively. WT and three independent transgenic lines were plated in per plate, with four seedlings of each genotype. The drought treatments in petri plate were performed five biological replicates. Scale bar: 1 cm. **c** Semi-quantitative RT-PCR analysis of *SpWRKY33* expression in WT and transgenic plants. **d** and **e** Statistics of root length and fresh weight of WT and *SpWRKY33*-overexpression plants, respectively. Values were means ± SD (*n* = 20). ** on the histograms indicates statistical difference between WT and transgenic plants at *P* < 0.01. **f** and **g** Phenotypic characteristics of WT and *SpWRKY33*-overexpression plants under normal water condition and withholding watering for 14 days, respectively. WT and three independent transgenic lines were grown in soil block, with four seedlings of each genotype. The drought treatments in soil were performed five biological replicates

Furthermore, we also investigated the role of *SpWRKY33* in drought tolerance using the same method. As shown in Fig. 7, three independent transgenic lines (OE-2, OE-3 and OE-8) with high abundance of *SpWRKY33* also exhibited increased drought tolerance. In the co-expression network, *SpWRKY33* was co-expressed with 15 genes (Additional file 8), and the expression patterns of their *Arabidopsis* homologous genes were compared between two-week-old *SpWRKY33*-overexpressing plants and WT plants. Among them, the expression levels of 13 genes were unambiguously detected; six genes were up-regulated, including *COR47* (2.1- to 2.7-fold), *EXL2* (2.4- to 2.7-fold), *HSPRO2* (1.7- to 2.3-fold), *VQ* (2.4- to 2.7-fold), *STP1* (2.4- to 2.9-fold), and *TPS9* (1.3- to 1.4-fold) (Additional file 2: Figure S7).

## Discussion

Accumulation of osmotic adjustment and enhancement of antioxidant enzyme activities are vital mechanisms of drought adaptation in plants [32, 33]. Osmotic adjustment through accumulating compatible solutes can promote water absorption from hypertonic solution or drought soils [34, 35]. Soluble sugars and proline are important compatible solutes, which regulate cell osmotic status, scavenge free radicals and protect cell membranes [33, 36]. Moreover, proline is proposed as a molecular chaperone functioning in stabilizing protein structure and as a component contributing to buffering of cytosolic pH [37, 38]. Reactive oxygen species (ROS) production is enhanced in cellular compartments under drought stress, and overproduction of ROS initiated uncontrolled oxidative cascades that damage cellular membranes and other cellular components and eventually cell death [39]. Antioxidant enzymes (e.g., POD, SOD, and CAT) have the ability to scavenge ROS and maintain redox equilibrium [40, 41]. Accumulation of osmotic adjustment and increase of scavenging enzyme activity have been detected during drought in wheat, maize, soybean, rice, potato, etc. [33, 39]. In our study, the findings of accumulation of soluble sugars and proline and enhancement of antioxidant enzyme activities suggested *S. psammophila* might resist drought through osmotic adjustment and ROS homeostasis to provide protection from cellular damage.

Besides of morphological and physiological adaptation, extensive transcriptomic reprogramming occurred to strive for survival under drought condition [42, 43]. Thus, understanding molecular mechanisms of drought response of *S. psammophila* is crucial to breed drought-tolerance willows. Based on the RNA-Sequencing profiling, we identified 8172 DEGs during drought stress in roots of *S. psammophila*, most of which enriched in multiple stress-related processes including transcriptional regulation, response to various stresses, cell death, protein phosphorylation and ubiquitination, ncRNA and DNA metabolic, etc. Thereinto, some processes have been confirmed as essential regulators or associated with plant tolerance to drought or other osmotic stresses. For instance, transcriptional regulation has been identified as an important regulatory mechanism of transcriptomic reprogramming in response to drought [10]. Transcriptional regulation was enriched in both clusters 2 and 3 (Fig. 4), and largest number of transcription factors existed in these two clusters (Additional file 2: Figure S4), suggesting this mechanism was dramatically evoked and executed function after short-term drought stress. Phosphoproteins have been identified under drought stress in several plants, and some proteins have been demonstrated to regulate drought tolerance via mediated phosphorylation [44, 45]. Furthermore, temporal patterns of the enriched GO terms showed that these biological processes occurred according to the time stages. These results revealed that comprehensive and well-ordered biological events motivated *S. psammophila* to resist and adapt to drought stress.

Based on whole-transcriptome datasets, WGCNA provides an effective approach to identify key regulators and it is widely used in various studies to explain molecular mechanisms of complex traits, such as growth, yield and stress-resistance. For instance, two potential key drivers for seed trait formation, *GA20OX* and *NFYA*, have been screened from co-expression network in soybean [46]. *WRKY40* and copper transport protein have been identified to be the hub genes involved in defense against whitefly infestation in cotton [47]. Three major hubs including *MYB2*, phosphoglycerate kinase and heat shock protein have been identified during salinity and drought stresses in rice [48]. In our study, we identified eight hub genes (*MDP-1, RGLG2*, *Mod*(*r*) *protein*, *CML8*, *RLCK*, *GRAM domain-containing protein*, *WRKY33*, and an unknown gene) that were co-expressed with a large number of genes in *S. psammophila*, suggesting their key positions in drought stress response. Through functional validation, two hub genes *SpMDP1* and *SpWRKY33* were proved to participate in plant drought tolerance. Thus, our study indicated that identification of candidate drought tolerance genes through co-expression network was highly efficient and credible.

As a member of haloacid dehalogenase superfamily, *MDP1* contains a conserved motif (Asp-X-Asp-X-Thr) [49], and its specific function in abiotic stress tolerance in plants remains unknown until now. In our study, *SpMDP1* had the highest connectivity with 380 genes, and its overexpression transgenic *Arabidopsis* could improve the tolerance to drought. This result provides new progress and evidence for *MDP1* involved in plant drought tolerance. Glycosylation is an important posttranslational modification of proteins, and it plays a crucial role in impacting protein functions and regulating biological processes [50]. For example, the *N*-glycosylation occurs in the proteins mainly involved in cell wall metabolism, stress response and proteolysis under drought in common bean [36]. Studies have demonstrated that MDP1 can cooperate with fructosamine-3-kinase to free proteins from the glycation products derived from Glu-6-P, indicating that *MDP1* may be the key component for glycosylation-involved protein repair [51, 52]. Whether the drought tolerance improved by *SpMDP1* is mediated by protein repair is needed to further research.

WRKY33 belongs to the evolutionarily conserved WRKY transcription factor. The expression of *WRKY33* is induced by pathogens and environmental stimuli, and its function in plant tolerance stresses has been confirmed through gene silencing and complementation [53, 54]. In *A. thaliana*, *atwrky33* mutants are compromised not only in plant tolerance to necrotrophic pathogens and *Botrytis cinerea* infection, but also in tolerance to salinity and heat stresses [53, 55, 56]. Complementation of *Arabidopsis atwrky33* mutant plants by tomato (*Solanum lycopersicum*) *SlWRKY33A* and *SlWRKY33B* can fully restore resistance to *Botrytis* and heat tolerance [57]. In this study, we demonstrated that overexpression of the hub gene *SpWRKY33* could enhance the tolerance to drought stress in transgenic *Arabidopsis* plants. Recent study has indicated that *AtWRKY33* interacts with two VQ proteins, SIGMA FACTOR-INTERACTING PROTEIN1 (SIB1) and SIB2, which act as activators of *AtWRKY33* in plant defence against necrotrophic pathogens [58]. Our study also identified a VQ motif-containing protein (*SapurV1A.0943 s0020*) was co-expressed with *SpWRKY33* in the co-expression network (Additional file 8), and its expression level was up-regulated in *SpWRKY33*-overexpressing plants (Additional file 2: Figure S7). Moreover, the analysis of genome-wide transcriptional responses to drought in two willow genotypes revealed that *WRKY33* has been identified as one of candidate genes with a putative function in drought response [18]. These results indicate that *SpWRKY33* is an ideal gene used on genetic engineering for the creation of new varieties with high tolerance to stresses in plants.

Except *SpMDP1* and *SpWRKY33* which were performed functional validation in this study, the other six hub genes might also participate in drought tolerance in plants. For example, *AtRGLG2* negatively regulates the drought stress response by mediating *AtERF53* transcriptional activity in *A. thaliana* [59]. The expression level of *AtCML8* is strongly and transiently induced by *Pseudomonas syringae* and overexpression of *AtCML8* confers on plants a better resistance to pathogenic bacteria compared to WT, suggesting that *AtCML8* plays a role in plant immunity [60]. Although there are no reports of function of *CML8* in drought tolerance; other members of the *CML* family, such as *Oryza sativa OsCML4* and *Solanum habrochaites ShCML44*, have been confirmed to enhance tolerance to drought stress in plants [61, 62]. Further studies are needed to investigate whether these hub genes contribute to drought tolerance in *S. psammophila*.

## Conclusions

In this study, we analyzed DEGs in *S. psammophila* roots under a time-course drought stresses, performed analysis of biological processes and pathways, and identified eight hub genes based on the co-expression network. We found the accumulation of osmotic adjustment substances and enhancement of antioxidant enzyme activities under drought stress in *S. psammophila*. Abundant DEGs were enriched in several stress-related processes including transcriptional regulation, response to various stresses, cell death, etc. Through overexpression in *A. thaliana*, two hub genes, *SpMDP1* and *SpWRKY33*, have been confirmed to enhance the drought tolerance in transgenic plants. Our results will contribute to parse the mechanism of drought tolerance of *S. psammophila* and facilitate identification of critical genes involved in drought tolerance for breeding willows.

## Methods

### Cultivation of *S. psammophila* and drought stress treatment

In our study, *S. psammophila* materials were obtained from the germplasm collection base of *S. psammophila* (E 110°38′59.1″, N 40°14′15.5″) in Ordos Dalad, Inner Mongolia. One-year-old twigs of *S. psammophila* were cut into 15 cm cuttings, water cultured in 1/2 strength Hoagland’s solution, and grown in a growth chamber under long-day conditions with 16 h light and 8 h dark at 24–25 °C. Ten-week-old seedlings were subjected to 22% (w/v) polyethylene glycol 6000 (PEG 6000)-simulated drought stress for four time points (0, 6, 24, and 96 h). At the end of each time point, fine roots were harvested, frozen immediately in liquid nitrogen, and stored in − 80 °C refrigerator for further analysis and sequencing. We performed three biological replicates.

### Measurement of physiological parameters of *S. psammophila* under drought stress

Electric conductivity (EC) of *S. psammophila* roots was measured by conductivity meter (DDS-307). Relative water content (RWC) was calculated as previous study [63]. The proline content was measured with ninhydrin method [64]. The soluble sugar content was measured with anthrone method [65]. For enzyme assays, superoxide dismutase (SOD) activity was determined by measuring its ability to inhibit the photochemical reduction of nitroblue tetrazolium at 560 nm [66], peroxidase (POD) activity was measured based on the change in absorbance of 470 nm due to guaiacol oxidation [67], catalase (CAT) activity was determined by monitoring the disappearance of H_2_O_2_ [68]. All experiments were performed three technical replicates, and the data were analyzed with Student’s *t* test.

### RNA isolation, library preparation, Illumina sequencing

Total RNA was extracted from roots using the RNeasy Plant Mini Kit (Qiagen, Germany) based on the manufacturer’s protocol. The RNA quality and quantity were detected by the NanoDrop system and Agilent2100 Bioanalyzer. The cDNA library was constructed and sequenced using Illumina HiSeq2500 platform by Biomarker Technologies Corporation (Beijing, China), with paired-end sequencing and read lengths of 125 bp. After quality-control procedure with filtering adapter sequences and low-quality reads, the clean data were obtained for subsequent analysis.

### Mapping and quantification of RNA-Sequencing data

We selected *S. purpurea* genome version 1.0 from Phytozome (https://phytozome.jgi.doe.gov/pz/portal.html#!info?alias=Org_Spurpurea) as reference genome, which is 392 Mb in size with 37,865 protein-coding loci and 61,520 protein coding transcripts. Clean reads were mapped to the reference genome with allowance for 5% mismatches using TopHat2 software [69]. Gene expression levels were calculated as reads per kilobase of transcript sequence per million base pairs sequenced (FPKM) using Cufflinks [70]. The differentially expressed genes (DEGs) were identified by DESeq [71]. A threshold of false discovery rate (FDR) ≤ 0.01 and an absolute value of log_2_ ratio > 2 were used to retrieve DEGs.

### Pathway analysis and functional categorization

To study the biological function of DEGs, gene set enrichment with gene ontology (GO) terms was performed using Blast2GO [72]. Metabolic pathways were analyzed using kyoto encyclopedia of genes and genomes (KEGG) program [73]. Fischer’s exact test was used to assess the significance of GO categories and KEGG pathways.

### Co-expression gene network generation

The co-expression network was analyzed using the weighted gene co-expression network analysis (WGCNA) R package [74]. The graphic network was created by Cytoscape 3.7.0 software [75].

### Quantitative real-time PCR (qRT-PCR)

Primer 3 software was used to design the primers, and qRT-PCR was performed according to the previous described method [76]. *SpUBC* (*Ubiquitin-conjugating enzyme E2*) and *AtActin* were used as the reference genes in *S. psammophila* [26] and *A. thaliana*, respectively. Four technical replicates and three biological replicates of each sample were performed. All of the primer sequences were listed in Additional file 9. For homologous genes identification between *Arabidopsis* and *Salix*, the BLASTp best hit of *Salix* genes from the co-expression network in *Arabidopsis* genome were used for qRT-PCR analysis in *Arabidopsis* transgenic lines.

### Plasmid construction and transformation in *A. thaliana*

The full length CDS sequences of *SpMDP1* and *SpWRKY33* were independently amplified from *S. psammophila* and cloned into pDONR222.1 for sequencing, and the correct coding sequences were sub-cloned into pMDC32 under driven of the cauliflower mosaic virus (CaMV) 35S promoter using Gateway system, respectively. Both above constructs were transferred into *A. thaliana* by floral dip method [77]. After screened on medium containing 25 mg·L^− 1^ hygromycin, more than 30 transgenic lines were obtained and the expression levels of these two target genes were detected using qRT-PCR.

### Drought tolerance analysis of overexpression plants

WT and homozygous T3 generation transgenic *Arabidopsis* lines were used in drought tolerance experiments. One-week-old sterilized seedlings were transferred into 1/2 strength MS medium containing 0 mmol/L (control) and 200 mmol/L mannitol (simulated drought stress), respectively. Ten days later, phenotypic traits were compared, and the root length and fresh weight of seedlings were counted. Drought treatment was also applied to three-week-old seedlings in soil with sufficient water by withholding watering for 14 days. Five biological replicates were performed to ensure results’ reliability, and data were analyzed by *t* test.

## Additional files

Additional file 1: Output statistics of RNA-Sequencing. (XLSX 11 kb)

Additional file 2: **Figure S1.** GO terms for molecular functions of DEGs during drought stress in *S. psammophila*. Only significantly enriched terms with corrected *P* < 0.05 were indicated. The color and size of each point represented the -log_10_ (FDR) values and enrichment scores. **Figure S2.** GO terms for cellular components of DEGs during drought stress in *S. psammophila*. Only significantly enriched terms with corrected *P* < 0.05 were indicated. The color and size of each point represented the -log_10_ (FDR) values and enrichment scores. **Figure S3.** KEGG enrichment analysis of DEGs during drought stress in *S. psammophila*. Only significantly enriched pathways with corrected *P* < 0.05 were indicated. The color and size of each point represented the -log_10_ (FDR) values and enrichment scores. **Figure S4.** Identification of 672 transcription factors (TFs) representing 45 gene families in the nine clusters. The color and size of each point both represented TF number. **Figure S5.** Identification of co-expression modules and their relationships. **a** Hierarchical cluster tree showing six co-expression modules identified by weighted gene co-expression network analysis (WGCNA). Modules corresponding to branches are labelled with colours indicated by the colour bands underneath the tree. **b** Cluster relationships of these six modules. **Figure S6.** Expression patterns of co-expression genes with *SpMDP1* in WT and transgenic plants. Expression patterns of up-regulated **(a)** and down-regulated **(b)** genes in *SpMDP1*-overexpressing plants. The expression level of each gene in the WT was set to 1. **Figure S7.** Expression patterns of co-expression genes with *SpWRKY33* in WT and transgenic plants. The expression level of each gene in the WT was set to 1. (PDF 1002 kb)

Additional file 3: GO enrichment analysis of all DEGs. (XLSX 82 kb)

Additional file 4: KEGG enrichment analysis of all DEGs. (XLSX 14 kb)

Additional file 5: GO enrichment analysis of nine clusters. (XLSX 45 kb)

Additional file 6: The genes in co-expression network. (XLSX 30 kb)

Additional file 7: The co-expressed genes with *SpMDP1. (XLSX 31 kb)*

Additional file 8: The co-expressed genes with *SpWRKY33. (XLSX 10 kb)*

Additional file 9: The primers of eight hub genes and co-expressed genes used in qRT-PCR analysis. (XLSX 12 kb)

## Data Availability

The RNA-Sequencing raw data have been deposited to the National Centre for Biotechnology Information (NCBI) BioProject database under accession number PRJNA485903. All the supporting data are included as additional files.

## Abbreviations

BP: Biological processes
CaMV: Cauliflower mosaic virus
CAT: Catalase
CC: Cellular components
CML8: Calmodulin-like 8
DEGs: Differentially expressed genes
EC: Electric conductivity
FDR: False discovery rate
GO: Gene ontology
KEGG: Kyoto encyclopedia of genes and genomes
LEA-HRGP: Late embryogenesis abundant hydroxyproline-rich glycoprotein
MDP1: Magnesium-dependent phosphatase 1
MF: Molecular functions
Mod(r): Modifier of rudimentary
PEG: Polyethylene glycol
POD: Peroxidase
qRT-PCR: Quantitative real-time PCR
QTL: Quantitative trait loci
RGLG2: RING domain ligase2
RLCK: Receptor-like cytoplasmic kinase
RWC: Relative water content
SIB1: SIGMA FACTOR-INTERACTING PROTEIN1
SOD: Superoxide dismutase
TFs: Transcription factors
UBC: Ubiquitin-conjugating enzyme E2
WGCNA: Weighted gene co-expression network analysis
WT: Wild type
