# Supplementary material for: Genome-wide transcriptomic analysis of a desert willow, Salix psammophila, reveals the function of hub genes SpMDP1 and SpWRKY33 in drought tolerance
Source: BMC Plant Biol. 2019 Aug 15;19:356. doi: 10.1186/s12870-019-1900-1 (PMC6694639; doi:10.1186/s12870-019-1900-1)
Supplement: Supplementary file 2 — Figure S1. GO terms for molecular functions of DEGs during drought stress in S. psammophila. Only significantly enriched terms with corrected P < 0.05 were indicated. The color and size of each point represented the -log10 (FDR) values and enrichment scores. Figure S2. GO terms for cellular components of DEGs during drought stress in S. psammophila. Only significantly enriched terms with corrected P < 0.05 were indicated. The color and size of each point represented the -log10 (FDR) values and enrichment scores. Figure S3. KEGG enrichment analysis of DEGs during drought stress in S. psammophila. Only significantly enriched pathways with corrected P < 0.05 were indicated. The color and size of each point represented the -log10 (FDR) values and enrichment scores. Figure S4. Identification of 672 transcription factors (TFs) representing 45 gene families in the nine clusters. The color and size of each point both represented TF number. Figure S5. Identification of co-expression modules and their relationships. a Hierarchical cluster tree showing six co-expression modules identified by weighted gene co-expression network analysis (WGCNA). Modules corresponding to branches are labelled with colours indicated by the colour bands underneath the tree. b Cluster relationships of these six modules. Figure S6. Expression patterns of co-expression genes with SpMDP1 in WT and transgenic plants. Expression patterns of up-regulated (a) and down-regulated (b) genes in SpMDP1-overexpressing plants. The expression level of each gene in the WT was set to 1. Figure S7. Expression patterns of co-expression genes with SpWRKY33 in WT and transgenic plants. The expression level of each gene in the WT was set to 1. (PDF 1002 kb) [file 12870_2019_1900_MOESM2_ESM.pdf]

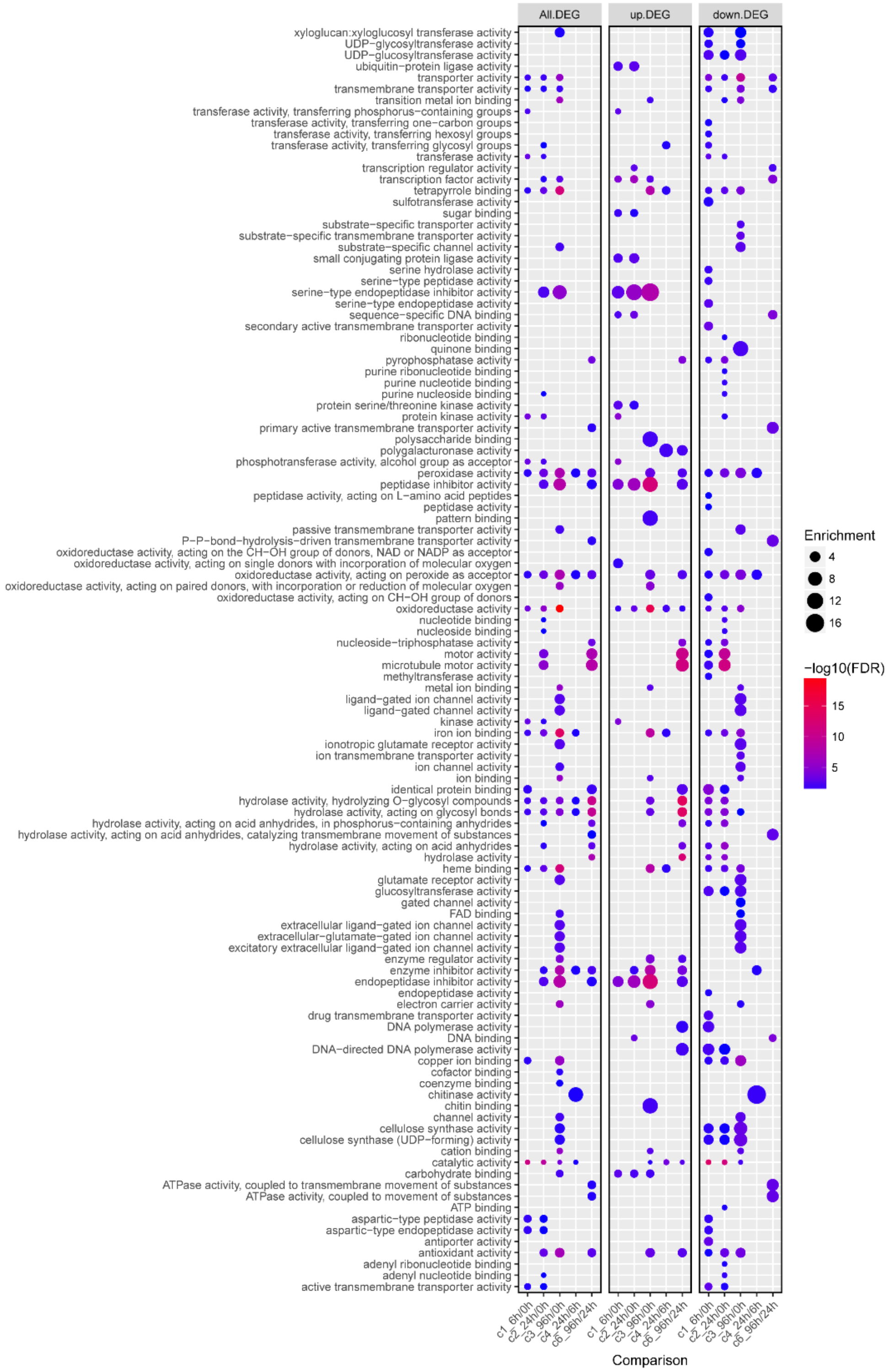

Figure S1. GO terms for molecular functions of DEGs during drought stress in *S. psammophila*. Only significantly enriched terms with corrected *P*-values < 0.05 were indicated. The color and size of each point represented the -$\log_{10}$ (FDR) values and enrichment scores.

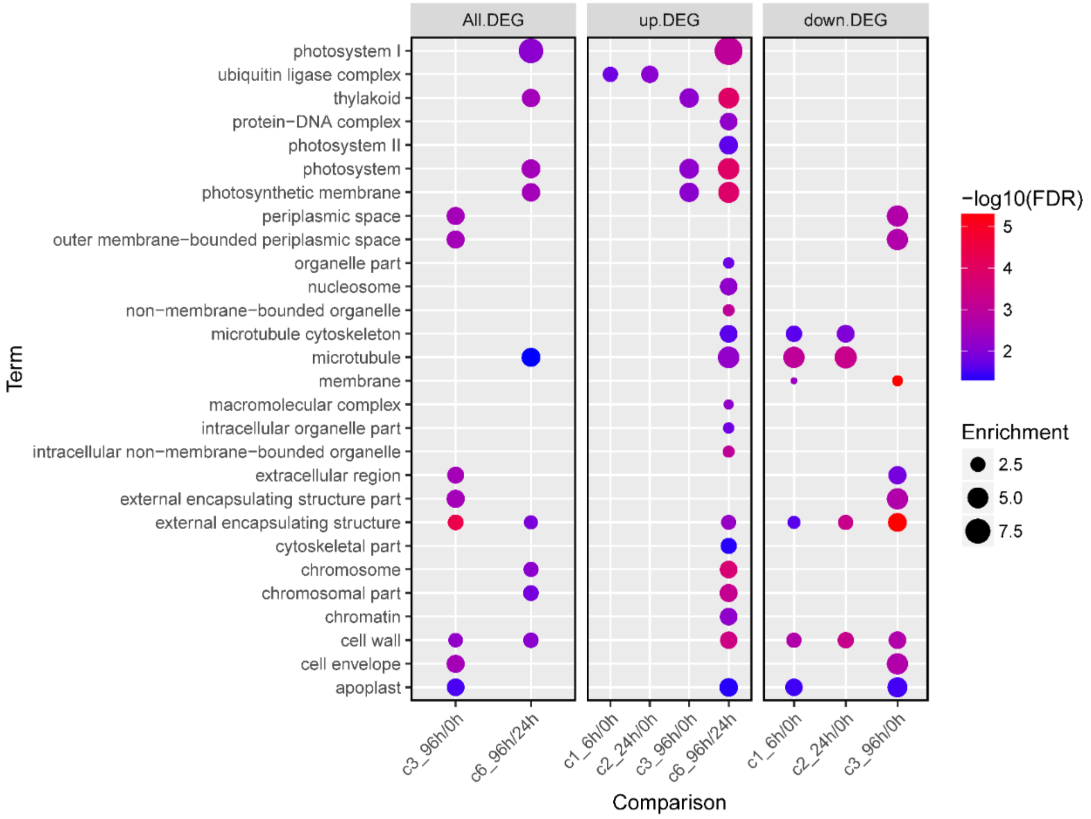

Figure S2. GO terms for cellular components of DEGs during drought stress in *S. psammophila*. Only significantly enriched terms with corrected *P*-values < 0.05 were indicated. The color and size of each point represented the -$\log_{10}$ (FDR) values and enrichment scores.

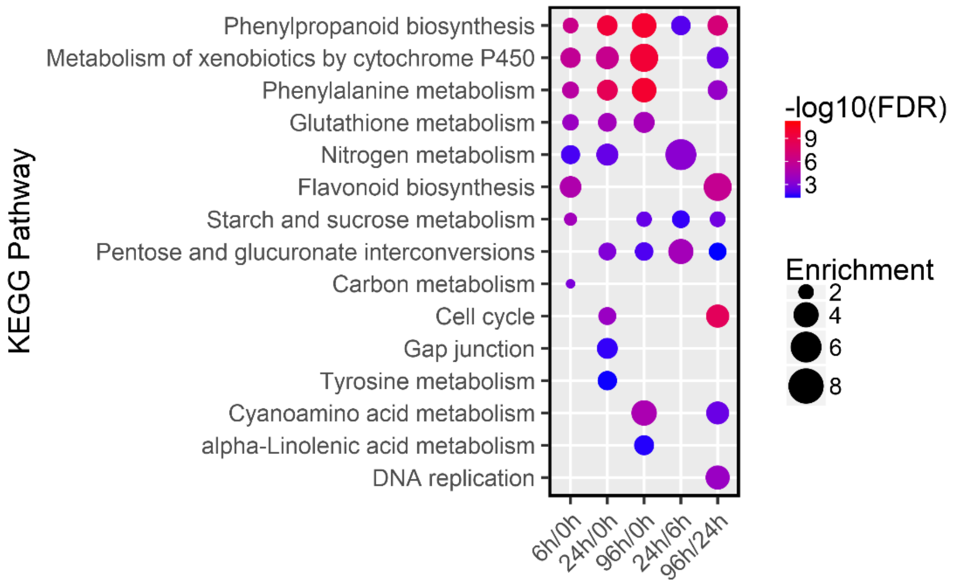

Figure S3. KEGG enrichment analysis of DEGs during drought stress in *S. psammophila*. Only significantly enriched pathways with corrected *P*-values < 0.05 were indicated. The color and size of each point represented the -$\log_{10}$ (FDR) values and enrichment scores.

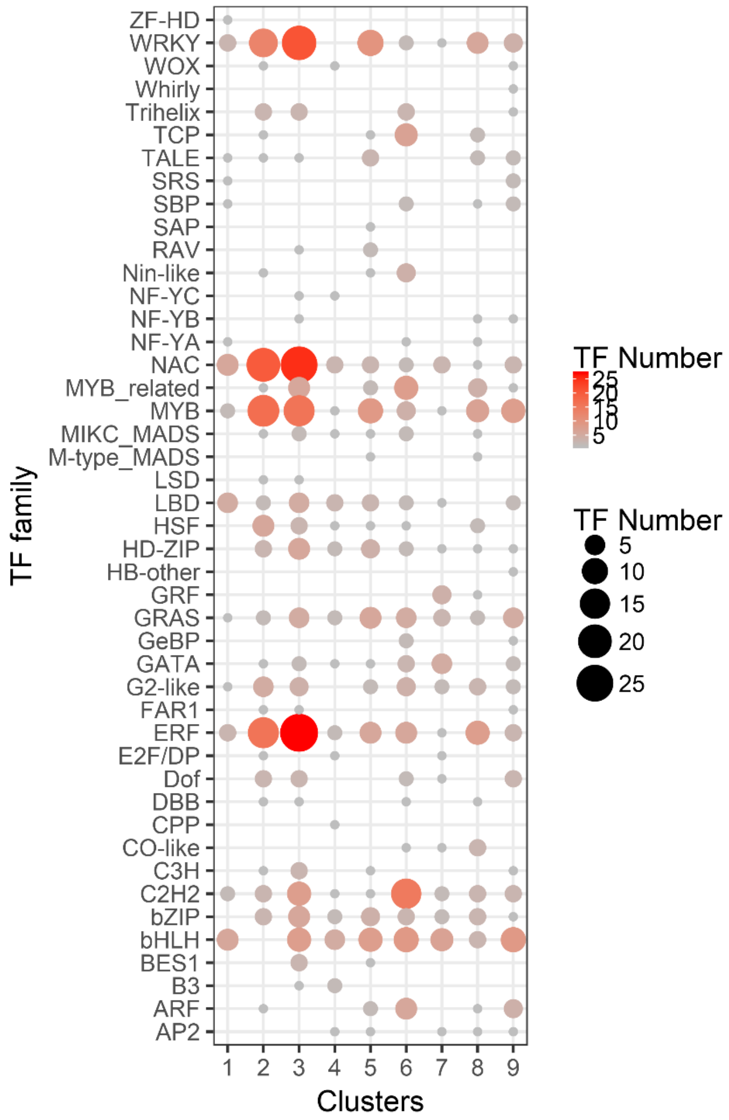

Figure S4. Identification of 672 transcription factors (TFs) representing 45 gene families in the nine clusters. The color and size of each point both represented TF number.

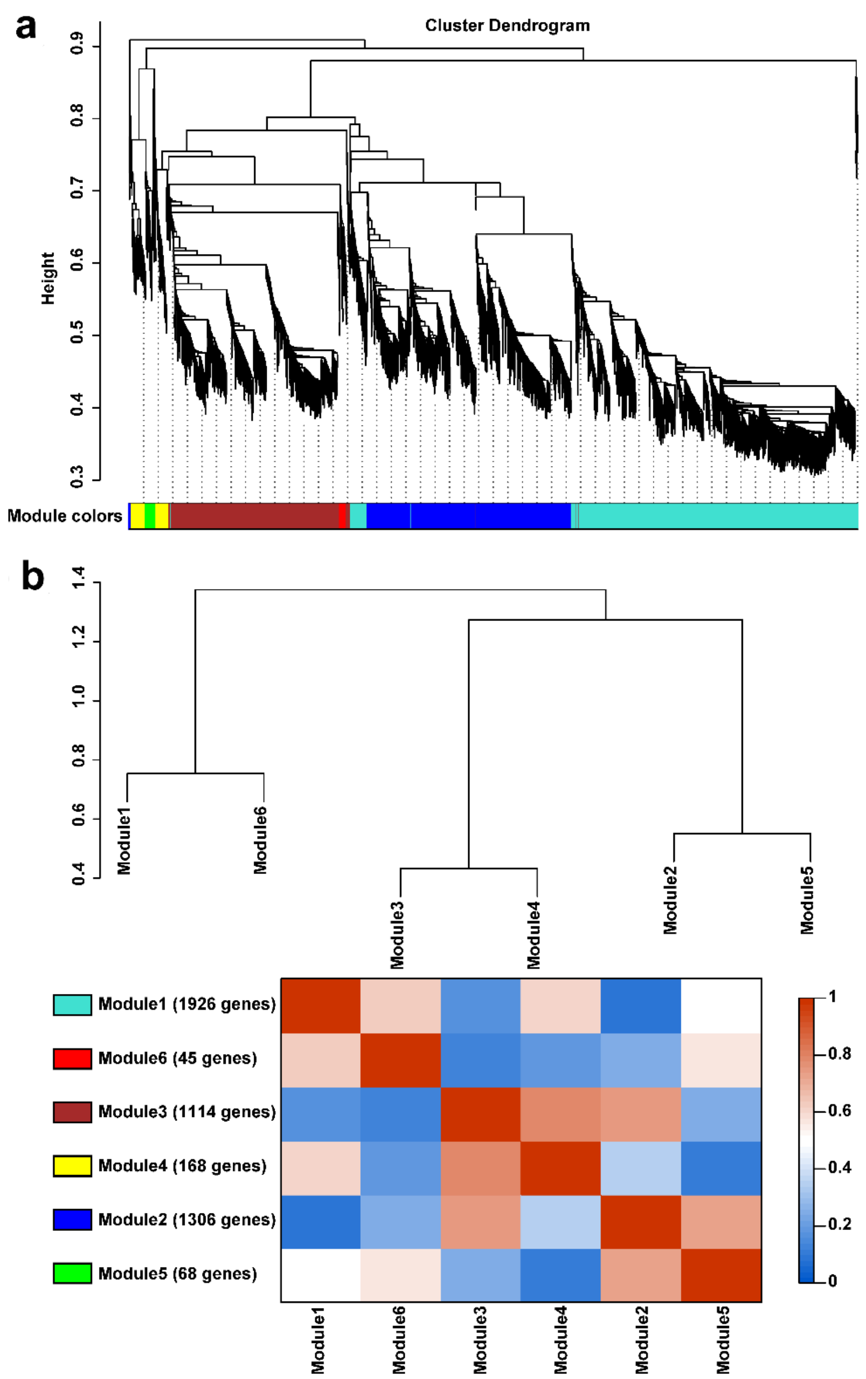

Figure S5. Identification of co-expression modules and their relationships. **a** Hierarchical cluster tree showing six co-expression modules identified by weighted gene co-expression network analysis (WGCNA). Modules corresponding to branches are labelled with colours indicated by the colour bands underneath the tree. **b** Cluster relationships of these six modules.

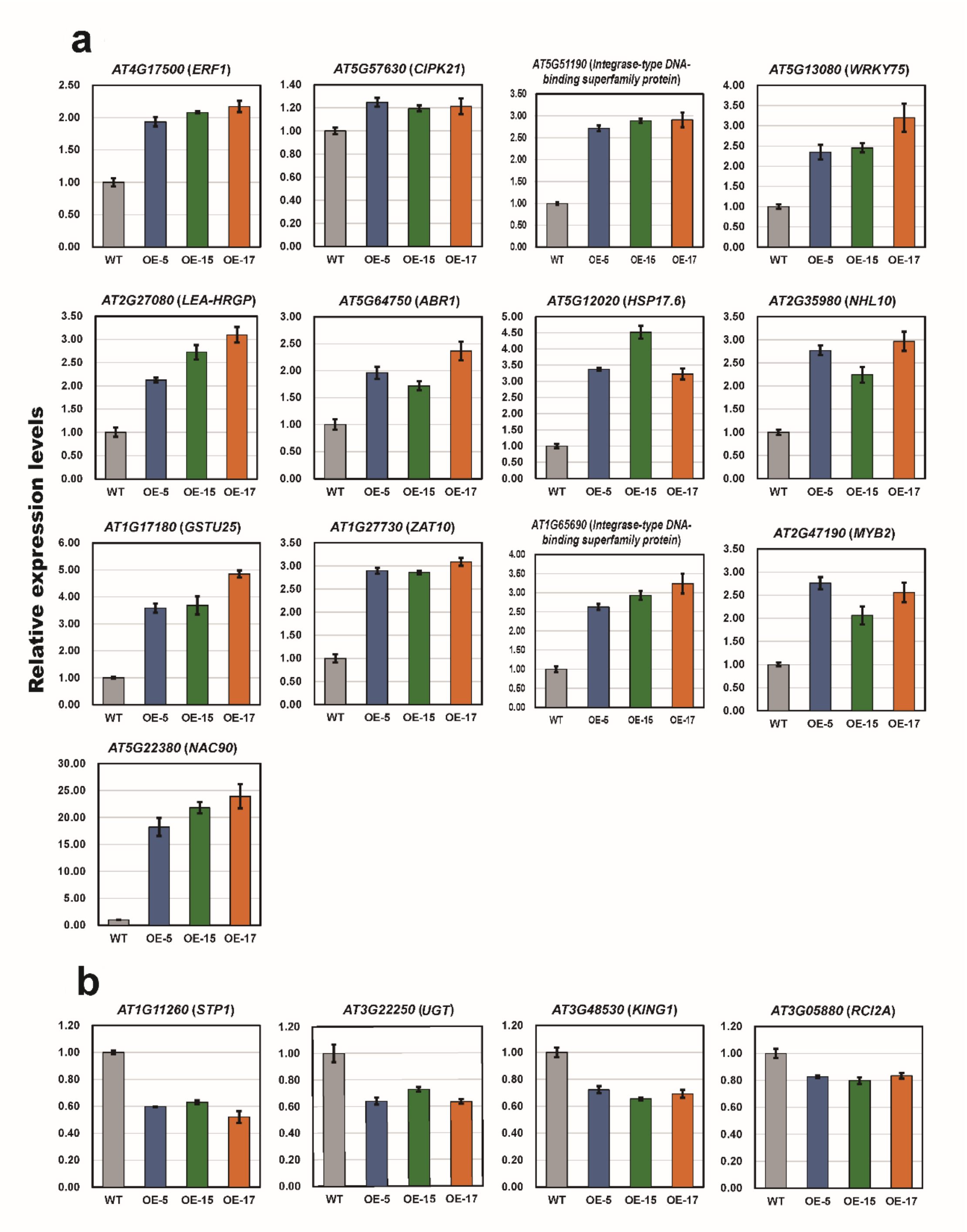

Figure S6. Expression patterns of co-expression genes with *SpMDP1* in WT and transgenic plants. Expression patterns of up-regulated **(a)** and down-regulated **(b)** genes in *SpMDP1*-overexpressing plants. The expression level of each gene in the WT was set to 1.

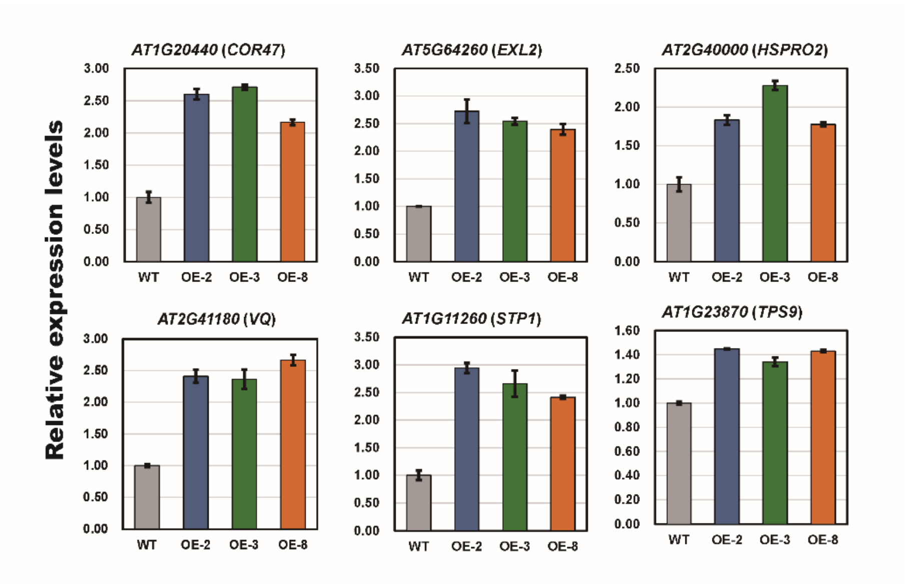

Figure S7. Expression patterns of co-expression genes with *SpWRKY33* in WT and transgenic plants. The expression level of each gene in the WT was set to 1.
